# A255 CHOLECYSTO-COLONIC FISTULA AND GASTRIC OUTLET OBSTRUCTION DUE TO EXTRINSIC COMPRESSION: A RARE BOUVERET-LIKE SYNDROME

**DOI:** 10.1093/jcag/gwac036.255

**Published:** 2023-03-07

**Authors:** A Kundra, P Mundra, S Srichandramohan, C Sypkes, N Calo

**Affiliations:** 1 Department of Gastroenterology, University of Ottawa, Ottawa; 2 McMaster University, Hamilton; 3 University of Ottawa, Ottawa; 4 Department of Medicine, University of British Columbia, Vancouver, Canada

## Abstract

**Background:**

Bouveret syndrome is a rare form of gastric outlet obstruction due to gallstone ileus. It is caused by passage of a large stone from the gallbladder into the duodenum via a bilio-duodenal fistula causing gastric outlet obstruction. It was first described by Leon Bouveret in 1896 and occurs most commonly in elderly patients with a higher incidence in women. In classic Bouveret syndrome, the stone itself becomes impacted in the intestinal lumen. A case series of 128 cases found only 2 cases where the endoscopic finding was of extrinsic compression.

**Purpose:**

To present a rare case of Cholecysto-colonic fistula and gastric outlet obstruction due to extrinsic compression as a rare Bouveret-like syndrome.

**Method:**

Chart review was conducted including clinical notes, laboratory, radiographic, and endoscopy reports. A relevant literature review was conducted.

**Result(s):**

A 76-year-old female with recent hospitalization for weight loss, anemia and cognitive decline presented to hospital with sudden onset vomiting without abdominal pain. She was admitted to general surgery. CT scan of the abdomen and pelvis were done and described a subhepatic inflammatory mass with inflammation of the proximal duodenum causing a partial gastric outlet obstruction as well as a fistula between with hepatobiliary system and a loop of ascending colon with associated pneumobilia. The etiology of the fistula was unclear but thought to be due to infection vs malignancy vs stone disease and the patient was put on antibiotic therapy. Due to inability to tolerate oral intake, she was put on parenteral nutrition. The Gastroenterology (GI) service was then consulted for consideration of gastroscopy and possible stent placement for obstruction. The GI service requested an MRI for further characterization. This revealed a 20mm impacted gallstone in the fistula tract with 3 other similarly sized stones passed into the colon, as well as evidence of a partial outlet obstruction due to the inflammatory mass in the porta hepatis. Gastroscopy was pursued to identify any intraluminal compression, which revealed duodenal edema in the first segment of the duodenum without any intraluminal pathology. Given that the compression was not intraluminal, a stent was not offered. Surgical options would be extensive surgery including cholecystectomy, duodenal wedge resection and partial colonic resection. Given her comorbidities, plan was made for conservative management instead. After 2 weeks of admission and conservative management, her outlet obstruction resolved and she was able to tolerate oral intake again.

**Image:**

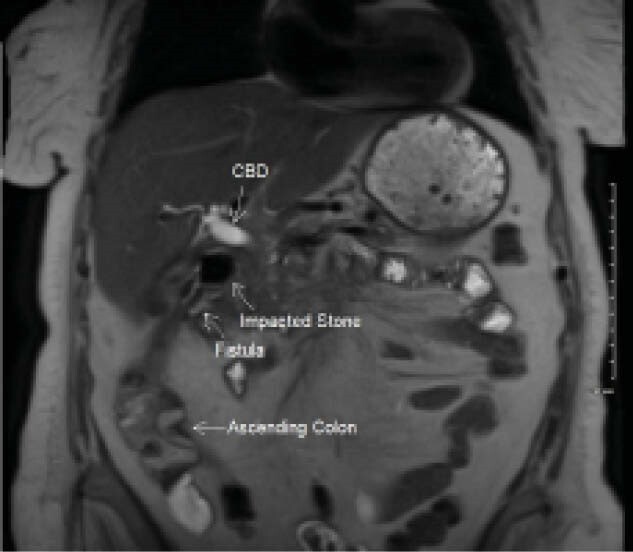

**Conclusion(s):**

Impacted gallstones causing chronic cholecystitis can result in fistula formation with the colon and due to the size of the stones in the tract and resultant inflammation, extrinsic compression of the duodenum may occur presenting as clinical gastric outlet obstruction. In this event, conservative management can be an effective strategy of management as a safe alternative to extensive surgical resection and bypass.

**Please acknowledge all funding agencies by checking the applicable boxes below:**

None

**Disclosure of Interest:**

None Declared

